# Surveillance of CTX-M group 1-producing *Enterobacterales* at the migratory Little Stint (*Calidris minuta)–*goat–aquatic interface in the Kenyan Rift Valley: a One Health approach

**DOI:** 10.3389/fmicb.2026.1893160

**Published:** 2026-09-15

**Authors:** Catherine W. Mbuthia, Rael J. Too, Alexanda Mzula, Anita Chepkemei, John Kiiru, Samuel Kariuki, Abubakar S. Hoza

**Affiliations:** 1 Department of Veterinary Microbiology, Parasitology and Biotechnology, College of Veterinary Medicine and Biomedical Sciences, Sokoine University of Agriculture, Morogoro, Tanzania; 2 Centre for Microbiology Research, Kenya Medical Research Institute, Nairobi, Kenya; 3 SACIDS Foundation for One Health, Sokoine University of Agriculture, Morogoro, Tanzania; 4 Department of Biological and Physical Sciences, School of Pure and Applied Sciences, Karatina University, Karatina, Kenya

**Keywords:** AMR, *Calidris minuta*, CTX-M group 1, Domestic goats, One Health Surveillance, PCR

## Abstract

The escalation of antimicrobial resistance (AMR) among critical-priority, extended-spectrum β-lactamase (ESBL)-producing pathogens poses a global public health threat. Applying a One Health framework, this study characterized CTX-M Group 1 gene variants alongside co-carried β-lactamase gene families (*bla*_OXA1 − like_, *bla*_SHV_, and *bla*_TEM_) in *Enterobacterales* across the migratory shorebirds Little Stints (*Calidris minuta*)*-*livestock-aquatic interface in the Kenyan Rift Valley. Sampling occurred across two migratory cohorts-arrival from the Arctic and pre-departure from the lakes in a repeated cross-sectional eco-surveillance design across two contrasting environments: Lake Bogoria (low anthropogenic pressure) and Lake Magadi (high pressure). From 389 fecal and water samples (*C. minuta, n* = 184; free-ranging domestic goats, *n* = 157; water, *n* = 48), 507 Gram-negative bacterial isolates were recovered, 479 of which were *Enterobacterales*. Phenotypic ESBL screening identified 15.7% (75/479) ESBL-positive *Enterobacterales* from 68 samples (*C. minuta, n* = 30; goats, *n* = 30; water, *n* = 8). Conventional polymerase chain reaction (PCR) of 79 isolates (75 phenotypic ESBL-*Enterobacterales* producers and four ESBL-negative *Klebsiella pneumoniae* controls*)* detected CTX-M Group 1 variants in 14 isolates (13 ESBL-positive strains and one ESBL-negative *K. pneumoniae*). The CTX-M Group 1 variants were identified in *Escherichia coli* (*n* = 6), *Enterobacter cloacae* (*n* = 4), *Klebsiella pneumoniae* (*n* = 3), and *Providencia rettgeri* (*n* = 1), frequently co-harboring *bla*_OXA1 − like_, *bla*_SHV_, and *bla*_TEM_ gene families. Detection frequencies exhibited site-specific and temporal trajectories across domains. To our knowledge, this is the first PCR-based detection of CTX-M Group 1 variants in *C. minuta* within this ecosystem. Rather than a mere passive corridor, the Kenyan Rift Valley serves as a dynamic ecological epicenter where global migratory flyways intersect with local anthropogenic drivers to modulate AMR. Despite the lack of sequencing data, this study provides vital baseline landscape data highlighting the need for sustained One Health-AMR surveillance and environmental stewardship.

## 1 Introduction

Antimicrobial resistance (AMR) is a paramount global health and socio-ecological crisis of the 21st century, profoundly impacting all domains of the One Health triad (Elbehiry et al., 2025). Driven largely by selective pressures from indiscriminate antimicrobial use (Christaki et al., 2019), resistance has rapidly emerged against highest priority critically important antimicrobials (HP-CIAs) for human medicine, notably third-generation cephalosporins (3GCs), such as cefotaxime (CTX), ceftazidime and ceftriaxone (Ribeiro et al., 2024). Consequently, the World Health Organization (WHO) classifies extended-spectrum β-lactamase (ESBL)-producing *Enterobacterales* as critical priority pathogens due to their ability to inactivate 3GCs and other β-lactams (World Health Organization, 2017).

The CTX-M, TEM, and SHV lineages constitute the primary determinants of ESBL-mediated resistance (El-Zayat et al., 2026). Within the CTX-M family, CTX-M Group 1 dominates, encompassing key clinical, veterinary and environmental variants including *bla*_CTX − M−1_, *bla*_CTX − M−3_, *bla*_CTX − M−15_ and *bla*_CTX − M−55_ (Husna et al., 2023). Foremost, among these is the *bla*_CTX − M−15_ gene, the globally dominant variant (Bevan et al., 2017). This challenge is amplified when ESBL-producers co-express multidrug resistance (MDR) to other critical drug classes, including fluoroquinolones, aminoglycosides, and macrolides (Taylor et al., 2021). Due to minimal sequence variation-for instance, *bla*_CTX − M−1_ and *bla*_CTX − M−15_ share ~98.7% nucleotide similarity, and differ by just one amino acid, conventional identification methods cannot easily distinguish specific variants (Higgins et al., 2023).

Once restricted to clinical settings, ESBLs epidemiology has expanded across environmental, livestock, and wildlife sectors (Zhang et al., 2025). Mobile genetic elements (MGEs) drive this dissemination via horizontal gene transfer (HGT) across hosts and bacterial genera (Partridge et al., 2018). While predominantly found in *Escherichia coli* and *Klebsiella pneumoniae*, promiscuous conjugative plasmids enable ESBL genes to spread to other *Enterobacterales*, including *Enterobacter*, Citrobacter, *Serratia, Proteus*, and *Salmonella* species (Dokuta et al., 2025; Ling et al., 2021).

Migratory wild birds are increasingly recognized as critical sentinels and transcontinental vectors in global AMR transmission cycle (Mbuthia and Hoza, 2025). By foraging in anthropogenic habitats contaminated with antimicrobial residues, avian hosts acquire resistant bacteria and disseminate them along migratory flyways (Chandler et al., 2020; Mbuthia and Hoza, 2025; Wu et al., 2018). Isolation of clinical resistance determinants from wild birds in remote regions like the Mongolian Gobi Desert highlights the global scope of environmental contamination (Guenther et al., 2017). Originating from the environmental genus *Kluyvera, bla*_CTX − M−15_ was amplified through high-risk clinical clones such as *E. coli* ST131 (Nicolas-Chanoine et al., 2014; Poirel et al., 2002) and mobile plasmids (Singh et al., 2022). The frequent detection of this variant in wild birds (Ben Yahia et al., 2018; Mohsin et al., 2017), makes it an ideal molecular marker for mapping ecological connectivity.

Additionally, livestock serve as stationary nodes for AMR maintenance (Ribeiro et al., 2024). Within commercial agricultural paradigms, intensive antimicrobials use in food-producing animals introduces AMR residues in environments, primarily through foodborne pathways or the deposition of contaminated livestock waste into soil and aquatic systems (Caneschi et al., 2023; Zalewska et al., 2021). However, in extensive, pastoralist systems, like the semi-arid Kenyan Rift Valley, transmission shifts from industrial inputs to fluid ecological interfaces. Lakes Magadi and Bogoria, hyper-saline-alkaline basins in southern and central northern Kenya, respectively (Pearson, 1987), and their surrounding scarce, freshwater sources represent critical points where livestock, wildlife and humans share the limited freshwater resources (Mbuthia et al., 2026). Beyond serving as shared drinking points, these aquatic environments act as foundational matrices for the selection and dissemination of AMR across biomes (Calero-Cáceres et al., 2022). Furthermore, the structural architecture of bacterial biofilms brings clinical, veterinary and environmental strains into close physical proximity, accelerating HGT of resistance determinants (Abe et al., 2020). Consequently, the shared fresh aquatic hubs within the two lake ecosystems may act as convergence zones for selection and circulation of bacterial resistance, particularly where organic waste and antimicrobial residues accumulate.

The Little Stint (*Calidris minuta*), a Palearctic migrant wintering in Kenya, relies on these freshwater sources while foraging on alkaline-saline mudflats of the two lakes (Mbuthia et al., 2026). Thus, these shared water sources serve as a vital bridge between local pastoral practices and global avian flyways.

Building on baseline phenotypic data, this study implemented a One Health molecular surveillance framework in the Kenyan Rift Valley. We targeted CTX-M Group 1 variants and co-carried β-lactamase gene families (*bla*_OXA1 − like_, *bla*_SHV_, and *bla*_TEM_) using polymerase chain reaction (PCR)-offering a scalable, cost-effective alternative for sequencing in resource-limited settings.

Surveillance spanned two migratory calendar periods across two distinct, scenic touristic landscapes. Lake Magadi- characterized by industrial trona mining, localized urbanization, severe freshwater scarcity and hyper-alkaline waters (pH 11.2)-restrict large-scale migratory avian staging. In contrast, Lake Bogoria (pH 9.0–10.2)-a protected Ramsar site, free from industrial extraction-serves as a quieter, highly connected flyway stopover for diverse resident and migratory bird species, drawn to its slightly restricted freshwater points (Mbuthia et al., 2026). Ultimately, this work illuminates how global flyways intersect with local land-use dynamics to drive AMR, providing a scalable framework for One Health antimicrobial stewardship across fluid interfaces.

## 2 Materials and methods

### 2.1 Study areas

Sampling of *C. minuta*, free-ranging domestic goats and shared freshwater sources was conducted across two alkaline-saline lakes in the Kenyan Rift Valley flyway. Lake Magadi (01° 55′ 0″ S, 36° 16′ 00″ E) is characterized by intensive trona mining, extensive pastoralism across the vast semi-arid dryland terrain, and low human population density (5–10 people per km^2^) in rural pastoral areas, with settlements concentrated around Magadi Town, the area's commercial hub. Peripheral freshwater hubs in this basin are heavily utilized by livestock, wildlife and local communities alike while the lake's warm spring pools are used for therapeutic bathing by the locals and tourists. In contrast, Lake Bogoria (00° 15′ 30″ N, 36° 06′ 00″ E), is a designated National Reserve and Ramsar site free from industrial mining. Settlements within its immediate perimeter are restricted, and the surrounding land-use is dominated by agro-pastoralism. Similar to Lake Magadi, peripheral freshwater points serve as key convergence zones for humans, livestock and wildlife (Mbuthia et al., 2026).

Figures 1A, B depict the sampling points at Lake Magadi and Lake Bogoria, respectively, and their surrounding freshwater points (Mbuthia et al., 2026).

**Figure 1:**
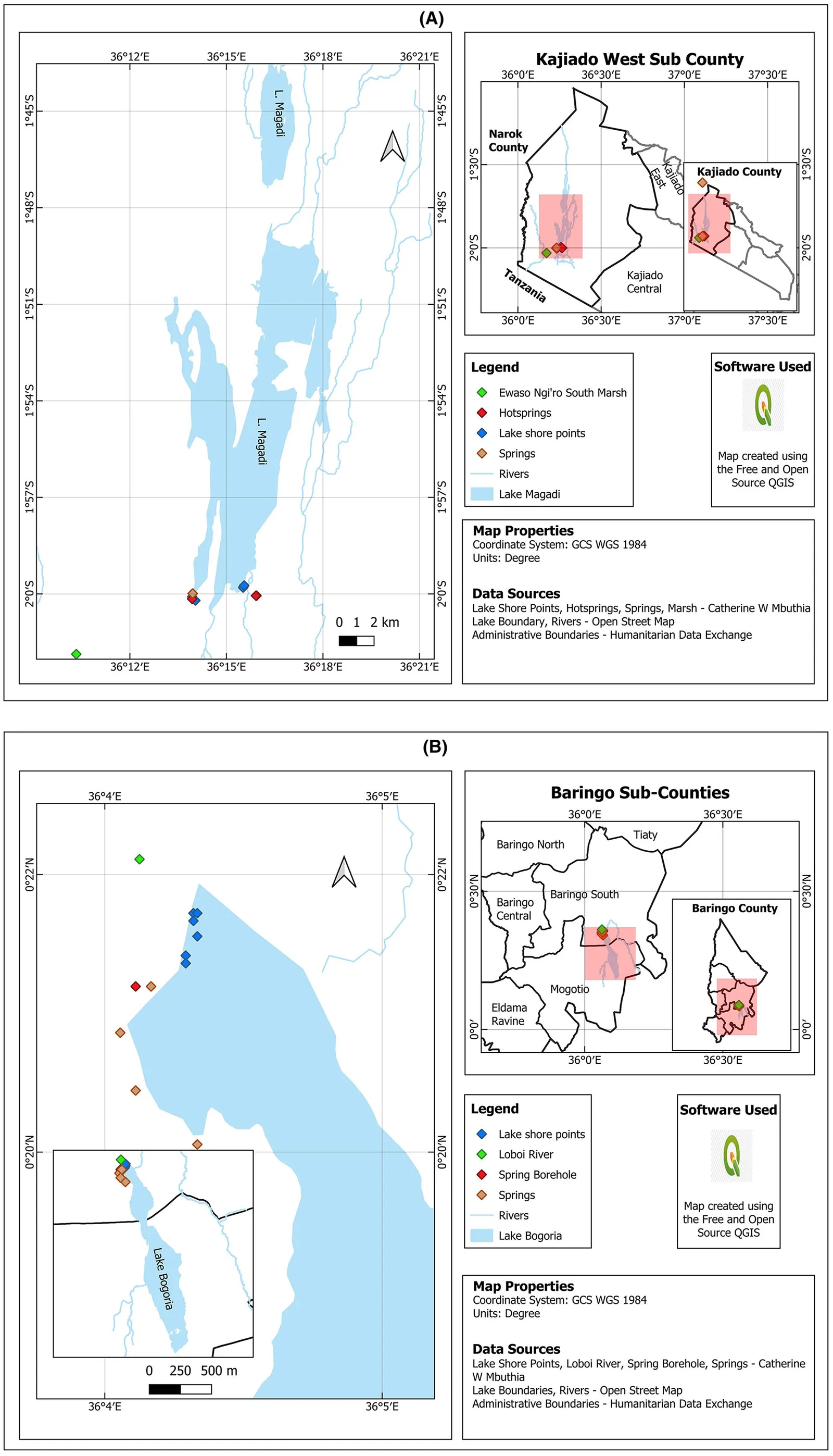
Spatial distribution of aquatic sampling points across the Kenyan Rift Valley. **(A)** Lake Magadi sampling network and associated freshwater hubs. **(B)** Lake Bogoria sampling network and associated freshwater hubs.

### 2.2 Study design, sampling, sample processing, and bacterial identification

This repeated cross-sectional study evaluated the eco-epidemiological dynamics of CTX-M Group 1-mediated ESBL-producing *Enterobacterales* across the migratory shorebirds (*C. minuta*)-livestock (domestic goats)-water interface. Samples were collected across two distinct migratory windows: arrival from the Arctic (Cohort 1: October 2021) and pre-departure from the lakes (Cohort 2: April 2022).

*C. minuta* were captured while foraging or drinking along lakeshores and peripheral fresh aquatic hubs using mist nets. To prevent resampling, each bird was fitted with a uniquely coded steel ring, held individually in a sterile ventilated cloth bag for non-invasive fecal collection, and released immediately. Goat fecal samples were collected alongside socio-demographic and epidemiological data gathered via structured questionnaires administered to consenting livestock owners. The questionnaires captured household metadata on livestock management, clinical and veterinary antimicrobial use, socio-economic status, and water, sanitation, and hygiene (WASH) dynamics. Collected *C. minuta* and goats fecal samples were preserved in Cary-Blair transport medium (Oxoid, Basingstoke, UK). Water samples (lakes, springs, artesian boreholes, marshes and inflowing rivers) were collected in sterile Facon tubes. All samples were stored and transported at 4 °C to the laboratory for processing within 72 h of collection.

Fecal samples (*C. minuta* and goats) were enriched in buffered peptone water at 37 °C for 18–24 h; and subcultured onto MacConkey (MAC; Oxoid, Basingstoke, UK) agar for the isolation of *Enterobacterales*. Water samples (50 mL) were processed via sequential vacuum filtration through cellulose membranes (41, 20, 10, 1.2, 0.8, and 0.45 μm; Nalgene™, Thermo Fisher Scientific) to manage clogging from turbidity. Membranes per sample were transferred into 50 mL MAC broth in sterile Whirl-Pak^®^ bags, homogenized, and incubated overnight at 37 °C, and subcultured onto MAC agar.

From each sample, 4–5 colonies representing distinct colony morphology and lactose fermenting phenotypes [lactose fermenters (pink colonies), late lactose fermenters (pale pink), and non-lactose fermenters (colorless)] on MacConkey were selected. Distinct pure colonies were then definitively identified using matrix-assisted laser desorption/ionization time-of flight mass spectrometry (MALDI-TOF MS; Bruker, Bremen, Germany).

### 2.3 Antimicrobial susceptibility testing and ESBL phenotyping

All *Enterobacterales* isolates were tested for antimicrobial susceptibility via the Kirby-Bauer disk diffusion method against 12 agents (Oxoid, Basingstoke, UK), representing eight antimicrobial classes: ampicillin (10 μg), amoxicillin-clavulanic acid (30 μg), cefepime (30 μg), cefotaxime (30 μg), ceftriaxone (30 μg), cefuroxime (30 μg), meropenem (10 μg), ciprofloxacin (5 μg), tetracycline (30 μg), chloramphenicol (30 μg), gentamicin (10 μg), sulfamethoxazole-trimethoprim (25 μg). Results were and interpreted according to Clinical and Laboratory Standards Institute guidelines. Phenotypic screening for ESBL production was performed using the double-disk synergy test (DDST) with amoxicillin-clavulanic acid and third-generation cephalosporins (cefotaxime and ceftriaxone; CLSI M100, 2024). MDR was defined as non-susceptibility to at least one agent in three or more antimicrobial categories (Magiorakos et al., 2012).

### 2.4 Molecular screening of CTX-M group 1 variants

#### 2.4.1 Isolate selection for CTX-M group 1 variants screening

A subset of 79 *Enterobacterales* isolates-comprising 75 phenotypic ESBL-producers and a random 50% subset (4/8) of the recovered ESBL-negative *K. pneumoniae* strains were selected for PCR screening of CTX-M Group 1 variants, *bla*_OXA1 − like_, *bla*_SHV_, and *bla*_TEM_ gene families.

#### 2.4.2 DNA extraction

Archived isolates were recovered from frozen storage (tryptic soy broth with 15% glycerol, Oxoid, Basingstoke, UK), subcultured onto MAC agar, and transferred to Mueller–Hinton agar (Oxoid, Basingstoke, UK), and incubated at 37 °C for 18–24 h. Genomic DNA was extracted using the crude cell lysate boiling method as previously described (Too et al., 2024). Briefly, distinct bacterial colonies were suspended in 200 μL of nuclease-free water, heated at 100 °C for 10 min in a thermal block, and centrifuged at 16,000 rpm for 3 min. The supernatant containing crude DNA was harvested and stored at −20 °C prior to downstream PCR analysis.

#### 2.4.3 PCR amplification

Conventional uniplex PCR was performed using gene-specific primers (Table 1) to screen for CTX-M Group 1 variants, as well *bla*_OXA1 − like_, *bla*_TEM_, and *bla*_SHV_ β-lactamase gene families. *Escherichia coli* strain *CC2*, an institutional repository isolate-previously confirmed by WGS to harbor *bla*_CTX − M−15_, *bla*_TEM − 1_, *bla*_SHV − 1_, and *bla*_OXA − 1_ determinants, served as the positive control, and nuclease-free water as the negative control. PCR amplifications were carried out in a 12.5 μL total reaction volume containing 8.5 μL nuclease-free water, 2.5 μL 5 × OneTaq^®^Master Mix (containing Taq DNA polymerase, deoxynucleotide triphosphates, magnesium chloride, potassium chloride, Tris-HCL buffer and stabilizers; New England Biolabs, USA), 0.25 μL of each forward and reverse primer (10 μM), and 1 μL of DNA template. Amplification was conducted in a Biometra TRIO thermal cycler (Analytik Jena, Germany) under the following conditions: initial denaturation at 95 °C for 30 s; 35 cycles of denaturation at 95 °C for 30 s, gene-specific annealing temperatures (Table 1) for 1 min, and extension at 68 °C for 1 min; followed by a final extension at 68 °C for 5 min.

**Table 1:** Primer sequences and PCR conditions for detection of ESBL and β-lactamase gene families.

Primer	Lineage/family/ subfamily	Primer sequence (5–3^′^)	Target genes	Amplicon size (bp)	Annealing temp (°C)	REF
CTX-M-15-F	CTX-M group 1	5**′**-GTG ATA CCA CTT CAC CTC-3**′**	CTX-M Group 1	255	61	Gharrah et al., 2017
CTX-M-15-R		5**′**-AGT AAG TGA CCA GAA TCA G-3**′**				
TEM-1-F	*bla*_TEM_	5′-GAT CTC AAC AGC GGT AAG-3	*bla*_TEM_	786	50	Gharrah et al., 2017
TEM-1-R		5′-CAG TGA GGC ACC TAT CTC-3′				
SHV-1-F	*bla*_SHV_	5′-ACT ATC GCC AGC AGG ATC-3**′**	*bla*__*S*_HV_	356	50	Gharrah et al., 2017
SHV-1-R		5′-ATC GTC CAC CAT CCA CTG-3**′**				
OXA-1-F	*bla*_OXA1 − like_	5**′**-GGC ACC AGA TTC AAC TTT CAA G-3**′**	*bla*_OXA1 − like_	820	62	Chepkemei et al., 2022
OXA-1-R		5**′**GAC CCC AAG TTT CCT GTA AGT G-3**′**				

bp, base pairs; temp, temperature; ref, reference.

PCR products (5 μL) mixed with 2 μL of 5 × loading dye (Thermo Fisher Scientific, USA) were resolved by horizontal agarose gels: 1% (w/v) for amplicons ≥500 bp and 2% for amplicons ≤ 500 bp in 1 × Tris-acetate-EDTA (TAE) buffer and stained with 2 μL of 10,000 × SafeView^TM^ Classic, DNA stain (Applied Biological Materials, Canada). Gels were run alongside a 1 kb DNA ladder (Accuris SmartCheck™, USA) and digitally documented using an Azure Biosystems Gel Doc system (USA).

### 2.5 Ethical approvals

#### 2.5.1 Animal and environmental ethical approvals

Ethical approvals and research licenses were granted by the National Commission for Science, Technology, and Innovation (NACOSTI), Kenya (Reference: 333849; License No: NACOSTI/P/21/13520), and the Directorate of Postgraduate Studies, Research, Technology Transfer, and Consultancy (DPRTC) at Sokoine University of Agriculture, Tanzania.

Field clearances and permits for the non-invasive sampling of wild birds (*C. minuta*) were issued by the Departments of Tourism and Wildlife of Kajiado and Baringo County Governments. Aquatic sampling permits were obtained from the respective County Departments of Water, Environment, and Natural Resources. Livestock sampling permits were granted by the respective County Departments of Agriculture and Livestock. Avian capture and release procedures were performed by qualified ornithologists, and no animals were injured or euthanized.

#### 2.5.2 Human ethics and informed consent

Structured questionnaires administration was approved under institutional clearances from NACOSTI and DPRTC. Written informed consent was obtained from all adult participants prior to questionnaire administration and livestock sampling. Community health workers provided oral translations of consent protocols in local languages when necessary and acted as witnesses to the consenting participants. All household and personal data were fully anonymized.

## 3 Results

### 3.1 Distribution of phenotypic ESBL-positive *Enterobacterales*

A total of 507 Gram-negative bacterial isolates were recovered from 389 fecal and surface water samples (*C. minuta, n* = 184; goats, *n* = 157; water, *n* = 48). Of these, 479 isolates were identified as belonging to the order *Enterobacterales*. Phenotypic screening via DDST confirmed 75 ESBL-positive Enterobacterales isolates recovered from 68 distinct samples (*C. minuta, n* = 30; goats, *n* = 30; water, *n* = 8), as certain samples yielded multiple ESBL-producing *Enterobacterale*s. Among the 75 phenotypic ESBL producers, 30.7% (*n* = 23) were non-MDR-ESBL producers while 69.3% (*n* = 52) exhibited an ESBL-MDR phenotype.

As illustrated in Figure 2, the phenotypic ESBL-producing *Enterobacterales* were dominated by *Enterobacter* spp. (*n* = 31); including *E. cloacae* (*n* = 27), *E. kobei* (*n* = 2), *E. bugandensis* (*n* = 1), and *E. hormaechei* (*n* = 1), followed by *Escherichia coli* (*n* = 17), *Klebsiella* spp. (*n* = 12; comprising *K. pneumoniae* (*n* = 9), *K. variicola* (*n* = 2) and *K. oxytoca* (*n* = 1). Other *Enterobacterales* included *Leclercia adecarboxylata* (*n* = 6), *Citrobacter freundii* (*n* = 6), and low-frequency species (*n* = 1 each) of *Raoultella ornithinolytica, Providencia rettgeri*, and *Pantoea eucrina*.

**Figure 2:**
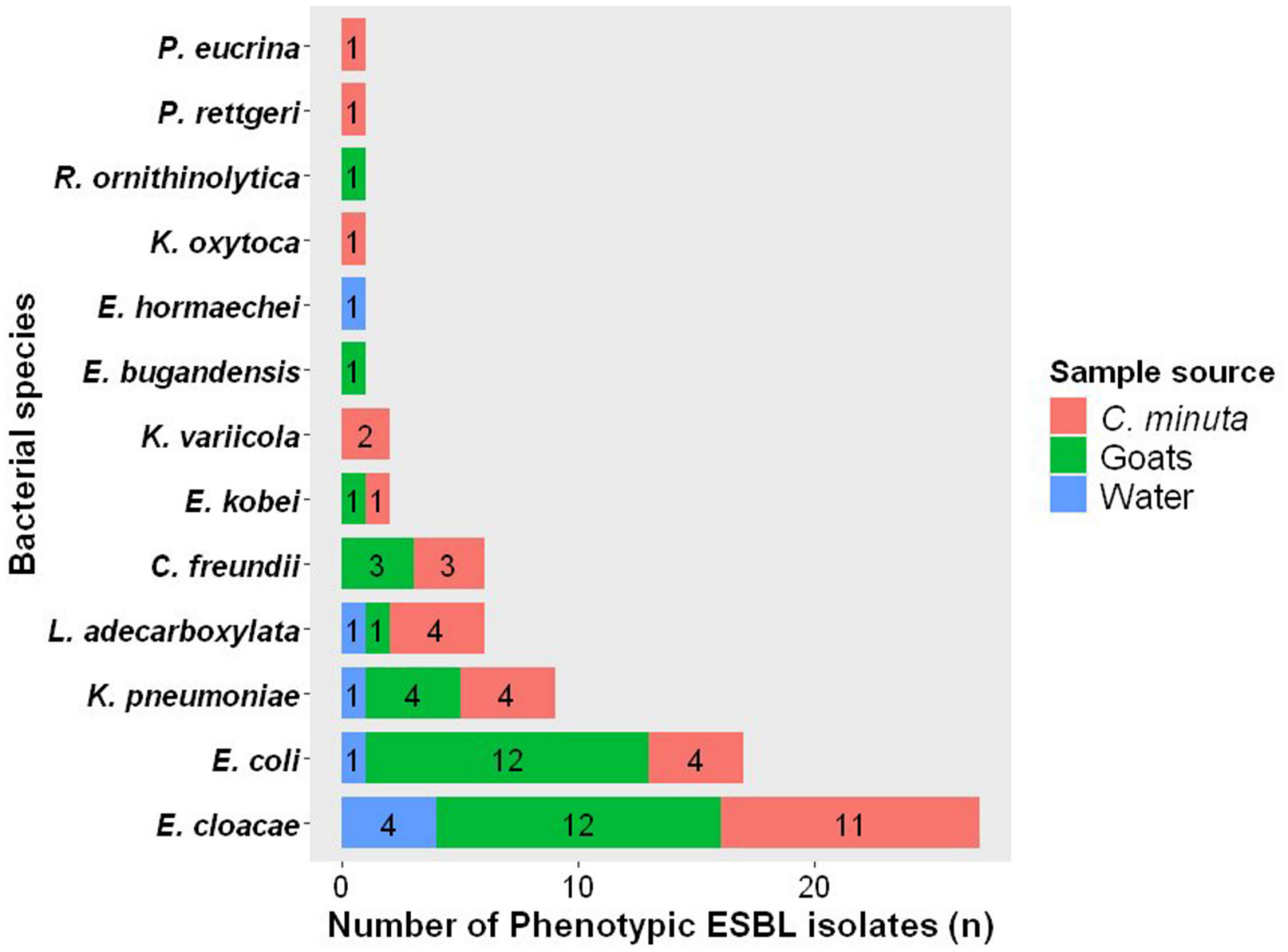
Distribution of phenotypic ESBL-producing *Enterobacterales* (*n* = 75) by bacterial species and sample source (*C. minuta*, goats and water).

### 3.2 Frequency and distribution of CTX-M group 1-positive *Enterobacterales*

PCR screening of the 79 selected *Enterobacterales* (75 phenotypic ESBL producers and four phenotypic ESBL-negative *K. pneumoniae* strains) confirmed the presence of CTX-M Group one variants in 14 isolates. These comprised 13 phenotypic ESBL producers and one phenotypic ESBL-negative *K. pneumoniae* strain (Supplementary Table S1).

Among the 14 CTX-M Group 1-positive isolates, *E. coli* (*n* = 6) was the predominant species, followed by *E. cloacae* (*n* = 4), *K. pneumoniae* (*n* = 3) and *P. rettgeri* (*n* = 1). Notably, one of the three CTX-M Group 1-positive *K. pneumoniae* carriers exhibited a phenotypically unexpressed ESBL profile (Figure 3).

**Figure 3:**
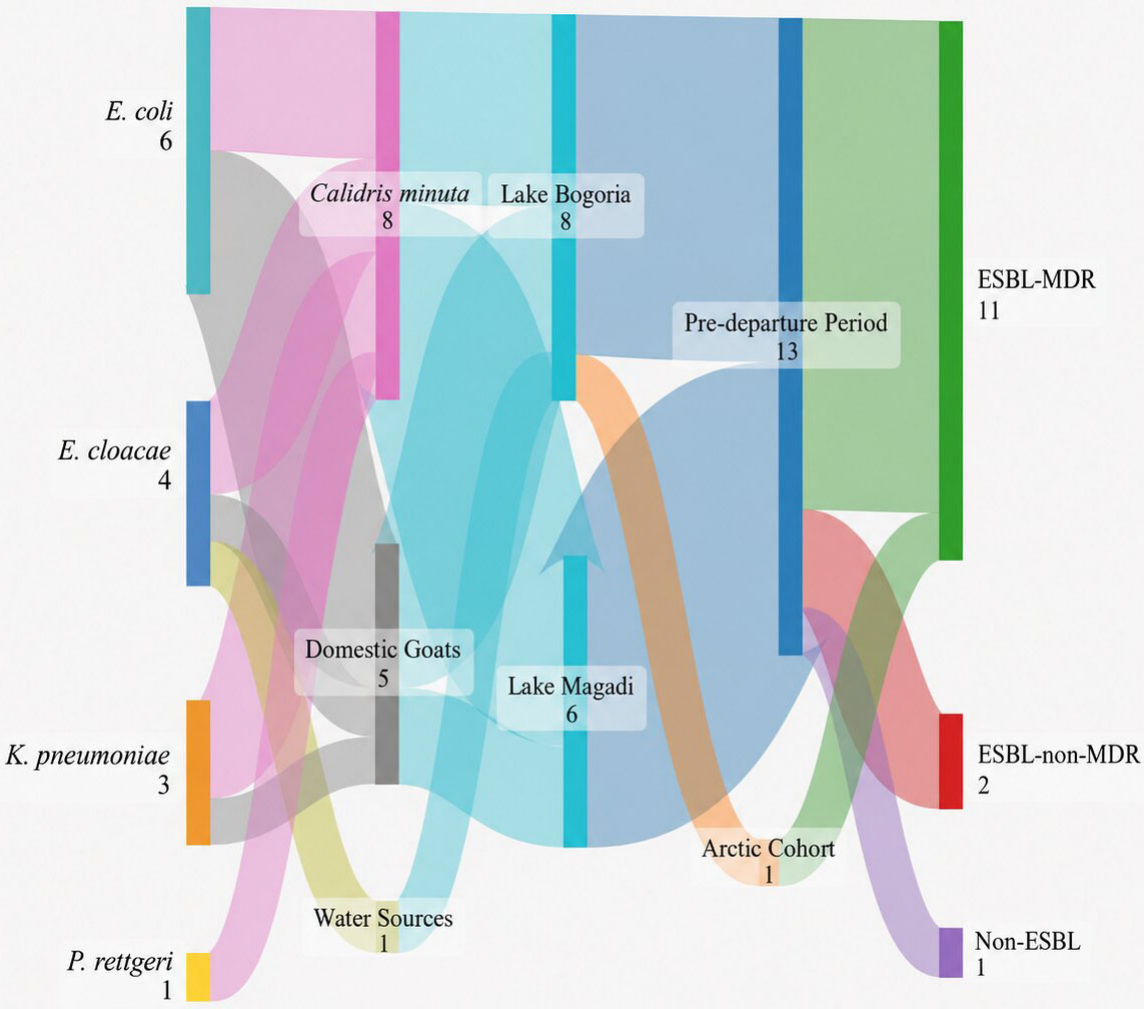
Multi-dimensional flow dynamics of CTX-M Group 1-positive *Enterobacterales* (*n* = 14) across Kenyan Rift Valley ecosystems (SankeyMATIC, 2026). Sankey diagram illustrating the interconnected distribution of CTX-M Group 1-positive bacterial species across host matrices (*C. minuta*, goats, and water sources), study sites (Lake Bogoria and Lake Magadi), sampling seasons (pre-departure and arctic cohort timelines) and phenotypic resistance profiles (ESBL-MDR, ESBL-non-MDR and non-ESBL). Nodes represent categorical variables, and connecting ribbon widths are proportional to isolate counts.

Across sample sources, CTX-M Group 1 was most prevalent in *C. minuta* (*n* = 8) followed by goats (*n* = 5) and water sources (*n* = 1): Lake Bogoria (*C. minuta, n* = 4; goats, *n* = 3; water, *n* = 1) and Lake Magadi (*C. minuta, n* = 4; goats, *n* = 2; water, *n* = 0; Supplementary Table S1).

Notably, all CTX-M Group 1-positive avian and livestock isolates originated exclusively from samples collected during the pre-departure period across both study sites. Conversely, the single CTX-M Group 1-positive water isolate was recovered from a freshwater hub at Lake Bogoria during the Arctic arrival window (Figure 3).

A comprehensive summary detailing the 79 *Enterobacterales* selected for PCR screening, their sample source, study site, phenotypic resistance profiles, CTX-M Group 1 distribution and co-carriage of β-lactamase gene families is provided in Supplementary Table S1.

### 3.3 Antimicrobial phenotypic co-resistance and genotypic co-carriage of CTX-M group 1-producers

Among the 14 CTX-M Group 1-positive isolates, 11 exhibited an ESBL-MDR phenotype, two showed a non-MDR-ESBL phenotype and one *K. pneumoniae* strain, was phenotypically non-ESBL (Figure 3). High levels of co-resistance were observed for ampicillin (*n* = 12), tetracycline (*n* = 10) and sulfamethoxazole-trimethoprim (*n* = 6). Resistance to CIAs was also detected, including gentamicin (*n* = 5), cefepime (*n* = 5), and ciprofloxacin (*n* = 2). Co-carriage analysis of β-lactamase gene families revealed *bla*_TEM_ in 9 isolates, *bla*_SHV_ in 1 isolate, and *bla*_OXA1 − like_ in 2 isolates (Supplementary Table S1).

## 4 Discussion

### 4.1 Migratory *C. minuta* and livestock as biological sentinels of AMR

The detection of CTX-M Group 1 variants in *Enterobacterales* isolates from migratory *C. minuta*, local livestock and shared aquatic environments underscore the necessity of integrated ecological surveillance across interconnected ecosystems. While PCR-based detection does not elucidate the directionality or mechanism of resistance transfer, the co-occurrence of identical resistance markers across taxonomic boundaries highlights the importance of monitoring multi-host interfaces within a One Health framework. Ultimately, these findings point to migratory shorebirds and local livestock as key co-occurring hosts and biological sentinels for AMR in the Kenyan Rift Valley. Our findings align with global research documenting the ubiquity of CTX-M Group 1 variants in wild birds across anthropogenic-influenced landscapes (Mbuthia and Hoza, 2025). However, the detection of these clinically critical genes in wild birds inhabiting remote areas (Guenther et al., 2017) indicates a broader epidemiological risk: migratory species may act as transboundary vectors, potentially transporting resistance determinants across vast geographical sites and bridging heavily contaminated regions with ecologically isolated environments (Shu et al., 2024).

### 4.2 Freshwater hubs as localized exposure bottlenecks

The landscape epidemiology of the Rift Valley reveals that aquatic architecture strongly influences CTX-M Group 1 distribution. The recovery of CTX-M Group 1 isolates restricted to multi-use freshwater hubs-an artesian borehole at Lake Bogoria and their complete absence in the hyper-saline-alkaline lakes uncovers a critical public health vulnerability. As the extreme alkalinity and salinity of the studied lakes create inhospitable conditions for most *Enterobacterales*, the persistence and potential dissemination of AMR are heavily concentrated within the peripheral freshwater hubs. Within these limited, high-density communal water points, sub-inhibitory concentrations of antimicrobial residues from direct fecal contamination by humans, livestock, and wildlife may converge creating favorable conditions for the co-selection and maintenance of resistant strains.

The ecological bottleneck is further highlighted by spatiotemporal contrasts across the study sites. The detection of CTX-M Group 1 in a freshwater artesian borehole at Lake Bogoria was restricted to the Arctic arrival sampling phase and was undetected during the pre-departure period. This temporal drop in open-water detection likely reflects hydrological flushing, solar ultraviolet (UV) inactivation, or the transition of resistant strains from planktonic water column into benthic sediment biofilms where environmental persistence typically occurs. Furthermore, the complete absence of CTX-M Group 1 variants in freshwater samples at Lake Magadi-despite its extreme freshwater scarcity-underscores that water limitation alone is insufficient to drive resistance concentration. Instead, Lake Bogoria's unique role as a highly connected migratory stopover attracts a broader, heterogeneous assembly of wild avian species, both migratory and resident. This seasonal avian influx could deliver a pulse of resistance elements that temporarily enriches the peripheral freshwater hubs where livestock, wildlife, humans and anthropogenic interfaces overlap.

### 4.3 Spatiotemporal drivers of AMR acquisition: anthropogenic factors and environmental fluctuations

The distinct spatiotemporal clustering of CTX-M Group 1 variants-detected exclusively in *C. minuta* and goats sampled during the pre-departure period-suggests temporal shifts in environmental resistance dynamics during migratory cycles. Four scenarios could explain this phenomenon. In the semi-arid Kenyan Rift Valley, limited water, sanitation and hygiene (WASH) infrastructure and unregulated human and veterinary antimicrobial pressure can create persistent local resistance pools, transforming shared freshwater points into exposure hotspots. Spatiotemporal clustering could also be modulated by local rainfall dynamics and avian physiological behavior. The pre-departure period (March–April) follows the prolonged dry season (January–February), during which peripheral freshwater points shrink significantly, forcing high-density overcrowding of pastoral livestock, humans, and wildlife around lingering water hubs. Concurrently, pre-departure shorebirds undergo intense pre-migratory fueling (hyperphagia)-dramatically increasing their water and food intake to build lipid reserves for their northward flight and this possibly accelerates exposure and horizontal gene acquisition at these concentrated aquatic bottlenecks. Additionally, pre-departure shorebirds have spent months foraging across human-impacted stopovers within the flyway, providing sufficient duration to acquire and harbor resistance traits prior to migration (“migratory baggage”).

Conversely, the absence of CTX-M Group 1 genes in newly arrived Arctic birds (sampled during October–November short rains) reflects both the ecological isolation of their high-latitude northern breeding grounds-where minimal human proximity limits exposure to anthropogenic resistance pools and the rain-driven expansion and dilution of local contaminated matrices. Rather than reflecting an inherent static trait of the flyway, this temporal contrast may indicate that the observed CTX-M Group 1 burden is strongly driven by localized, duration-dependent environmental exposures and seasonal hydrological contraction at key stopover sites.

### 4.4 Methodological implications for eco-surveillance: molecular vs. phenotypic concordance

From a surveillance-design perspective, our findings highlight a notable constraint in standard phenotypic monitoring protocols. To ensure robust surveillance across the migratory bird-livestock-water nexus, our PCR screening extended beyond phenotypically resistant ESBL-strains to include a subset of phenotypic ESBL-negative *K. pneumoniae* isolates. Crucially, owing to the clinical significance of *K. pneumoniae* as a high-priority ESKAPE pathogen in hosting and transferring MDR plasmids (Navon-Venezia et al., 2017), this phenotype-independent approach allowed us to evaluate phenotypically unexpressed resistance determinants.

Notably, a single goat *K. pneumoniae* isolate harbored CTX-M Group 1 variants, despite exhibiting an ESBL-negative phenotype. While a single observation cannot define population-level frequencies of silent carriage, it illustrates that resistance determinants can remain phenotypically unexpressed under standard screening conditions. Moreover, instances where a resistant phenotype lacked targeted β-lactamase gene families reflect the inherent specificity limits of candidate-gene PCR panels. These individual discrepancies highlight the necessity of high-resolution genomic approaches-such as whole-genome sequencing (WGS) to resolve gene expression dynamics and identify uncharacterized resistance determinants.

### 4.5 Background: mobile genetic elements and co-selection mechanisms

Mobile genetic elements (MGEs)-particularly conjugative IncF plasmids, *ISEcp1* insertion sequences and *ISCR1* elements*-*are well documented drivers of CTX-M genes mobilization and MDR cassette consolidation across host species (Gonullu et al., 2008; Kiiru et al., 2013; Woodford et al., 2009). While the specific MGEs were not sequence-characterized in the present study, these established mobilization mechanisms provide plausible context for the high levels of phenotypic co-resistance observed in our CTX-M Group 1-positive isolates to non-beta lactam classes (tetracycline, sulfamethoxazole-trimethoprim, gentamicin and ciprofloxacin), alongside expected beta-lactam resistance (ampicillin).

### 4.6 Limitations and recommendations

Despite these insights, this study is constrained by specific methodological limitations. Methodologically, our protocol relied on MALDI-TOF MS, antimicrobial phenotypic screening and targeted conventional PCR, without the benefit of WGS to map clonal lineages across the interfaces. Furthermore, we did not employ long-read or targeted amplicon sequencing to perform plasmid typing and resolve the fine-scale genetic architecture of CTX-M Group 1 gene cassettes. Additionally, the lack of environmental residue measurements leaves local selective pressures unquantified, while the absence of formal statistical testing limits risk-factor evaluation, and the lack of satellite telemetry prevents confirming the exact flyways and stopover histories of the sampled birds.

To transition effectively from passive monitoring to predictive eco-surveillance, future research must prioritize an integrated, multi-sectoral approach to address these gaps directly. Mechanistically, this requires the deployment of WGS and targeted amplicon sequencing, to resolve high-resolution genomic architecture and map HGT dynamics across One Health interfaces. Incorporating chemical profiling to quantify local antimicrobial residue concentrations will also be vital to establish baseline selective pressures in aquatic environments. Pairing these genomic insights and environmental residue profiling with satellite telemetry and real-time longitudinal fecal sampling is essential to validate the “migratory baggage” hypothesis and pinpoint international stopover sites where migratory birds acquire high-priority resistance genes.

Ultimately, the operationalization of these scientific insights demands a fundamental shift in governance and environmental policy. National action plans (NAPs) for AMR must be updated to institutionalize eco-surveillance, formally moving wildlife and environmental monitoring from the periphery into state-funded, systemic surveillance frameworks. Furthermore, robust antimicrobial stewardship must extend beyond clinical setting to encompass proactive landscape interventions at critical points of convergence. Local authorities should prioritize protecting vulnerable freshwater hubs by upgrading WASH infrastructure to attenuate the local selective pressures. By executing these high-impact, transdisciplinary interventions at aquatic points where host populations collide, we can effectively disrupt AMR transmission pathways across the One Health continuum, ultimately safeguarding both local pastoral food security and global public health.

## 5 Conclusion

The presence of CTX-M Group 1 variants in *C. minuta* highlights the potential role of migratory shorebirds as mobile global sentinels for monitoring environmental dissemination of critical AMR determinants. Within the semi-arid Kenyan Rift Valley, scarce freshwater resources create severe ecological bottlenecks, where intercontinental wild avian flyways intersect with pastoral livestock and human activities. The recovery of shared resistance profiles across these distinct domains suggests high ecological connectivity, positioning communal freshwater points as critical nexuses for potential resistance exchange. Furthermore, detecting CTX-M Group 1-producing pathogens at the wildlife-livestock-aquatic interface underscores the expansion of priority resistance markers into non-clinical settings By documenting these shared resistance traits across overlapping domains, this study reinforces the interconnected nature of resistance dynamics within a One Health framework and emphasizes the urgent need to integrate wildlife and the environment into national AMR surveillance and stewardship strategies.

## Data Availability

The datasets presented in this study can be found in online repositories. The names of the repository/repositories and accession number(s) can be found in the article/Supplementary material.
